# Academy of Stomatology

**Published:** 1897-05

**Authors:** 

**Affiliations:** The Academy of Stomatology


### ACADEMY OF STOMATOLOGY.

A regular meeting was held by the Academy of Stomatology January 26, 1897, at its rooms 1731 Chestnut Street, the President, Dr. James Truman, presiding.

The Clinic Committee reported two clinics held the previous Saturday. One by Dr. H. E. Roberts upon novel and quickly-constructed orthodontic appliances; the other by Dr. M. H. Cryer, who exhibited a case for whom he had performed a surgical operation to facilitate the moving of a malposed cuspid tooth.

The council reported the following acknowledgment of a valuable gift of books to its library by the family of the late Professor J. E. Garretson:

The Academy of Stomatology extends to the family of the late Professor Garretson a vote of thanks for the valuable contribution of books, presented in the name of his grandson, Edmund Garretson Cooke.

The Academy of Stomatology has satisfaction in taking advantage of the opportunity to express its high appreciation of the character of Dr. Garretson and of his abundant, honorable, and advanced work in the field of oral surgery in which he was eminent.

The Academy has further pleasure in devoting the evening of January 26, 1897, to a review of the life work of Dr. Garretson.

(Signed) Louis Jack,
Henry H. Burchard,
Robert Huey,
S. H. Guilford,
M. H. Cryer,
*Council.*

*The President.*—The subject of the evening will be opened by an essay on "The Work of James Edmund Garretson, A.M., M.D., D.D.S.," by Dr. Burchard.

(For Dr. Burchard's essay, see page 151.)

*The President.*—Gentlemen, you have heard this able and appreciative essay on Dr. Garretson's life work. Remarks are now in order, and I will call upon Dr. Guilford to open the subject.

*Dr. Guilford.*—Mr. President and Fellow Members,—I had not conceived the idea of speaking upon the subject this evening, because I have already spoken and written on it. However, while listening to the paper of the evening a few remembrances recur to me, which illustrate the character of Dr. Garretson.

All who knew Dr. Garretson were impressed with certain qualities he possessed,—which were characteristics. In the first place, he was a sincere man,—absolutely, conscientiously sincere. In the second place, he was an earnest man. He never did anything in his life, I believe, but what he put his whole soul in it. No one who ever knew him doubted his sincerity. He never pretended to love a man, or to care for one, without really doing so. He never flattered any one; he often praised men, praised them to their faces, and he meant it. He was not lavish in his praise; in fact, on many occasions it was thought that he withheld praise where it was due, but when he did give it he meant it. He was so earnest that whatever he did he did with a certain degree of solemnity. He was fond of humanity. He appreciated a joke as much as any one, and yet at the same time in everything that he did there was an earnestness that impressed those about him. I suppose it was due to the fact that he was absorbed in his own contemplations. These qualities, earnestness and sincerity, commanded the respect of all who knew him.

He possessed a power to attract, not only those of his own age, but younger people, particularly young men. His influence over young men was remarkable, he influenced them because he loved them, and they felt that he was always ready to listen to anything they might confide in him. They went to him for advice and they got it; they went to him for sympathy in their troubles and he gave it to them. If they needed more material aid he was always ready to assist them. He was a remarkably charitable man,—charitable in giving material help, as well as in other ways, and yet he knew the value of money. For many years he was a demonstrator in one of our colleges; after laboring there half a day he would wash his hands and go out and eat a ten cent lunch, and yet when he came to die he had been so prudent that he had accumulated a competence.

I remember a case of destitution in a little village where I lived that was particularly pathetic in its details; one that appealed to the sympathy of all those who knew of it, and there were very few who did. I wrote a few notes to some friends in regard to getting together a fund to assist the family. I wrote to Dr. Garretson and told him that I had requested a few of my friends to contribute five dollars apiece. He wrote back enclosing ten dollars and saying, "My dear Doctor,—Don't speak about $5.00. Let them give $10.00, and if more is needed call on me." Now, that showed the character of the man. It was this kindness of heart that attracted men to him. He not only appeared to but actually did sympathize with all who placed their confidence in him, and it was for this reason that he was beloved by so many people, particularly by the young people. I do not know that he had a single enemy. I never heard of one. I never heard a harsh or an unkind word said of him after his death; nothing but praise from every one, not so much because they appreciated his greatness in his own particular line, but because they appreciated his greatness of heart, one of his chief characteristics.

The President next called upon Dr. Cryer.

*Dr. Cryer.*—For eighteen years I followed that gentleman day after day and loved him as I loved no other man except my father and brother, and whenever I think of him and hear him mentioned it overcomes me and I cannot speak upon this subject.

The President called upon several gentlemen, who did not respond.

*Dr. James Truman.*—I should like to express briefly my feelings in regard to Dr. Garretson. I do not think the Society should pass this subject without more remarks upon a man who has done so much to elevate dentistry. We not only appreciate this part of his life work, but also that performed for surgery. He opened new lines of thought and spread abroad new ways of doing or performing surgical operations, which I think must have a lasting effect upon the medical and surgical practice. I do not know that they recognize it, but to my mind his contributions are a great force.

Dr. Garretson and myself were personal friends. We never met but it was a pleasant reunion. I enjoyed the conversation we had together on many subjects, philosophical and otherwise. I do not think as a man he was understood. I fear that when he came before the public as a speaker, his remarks were not fully comprehended. He talked generally in a language above that used by the common people, but when his thought could be absorbed and followed to legitimate conclusion, it exhibited a vital force not usual in public efforts.

I shall never forget, so long as life lasts, his address on the occasion of the Well's Memorial Meeting. It struck me as being one of the most forcible, as well as one of the most powerful, addresses that I had ever listened to, and yet I fear the majority of that audience did not fully appreciate it. There is something in becoming acquainted with a man's mode of speech in order to comprehend an address of this character. I sat under Dr. Garretson in his philosophical lectures during one entire course, therefore I became more familiar with his mode of thought, and probably better able on that account to reap the value of his ideas. He, to my mind, was one of the great men in dentistry, and we are here to-night not so much to mourn his loss as to remember him in his work.

### INCIDENTS OF PRACTICE.

*Dr. Jack.*—I wish to present the report from the notes of the conservative treatment of a pulp which I believe to be the first attempt of the kind made by me. The date was in September of 1866. The surface of decay the occlusal. The condition one of evident exposure to the carious denture, the last layers of which were not removed. A concave cap made of a heavy mat of gold foil was filled with a paste composed of hypophosphite of lime and glycerin; this, being laid over the part, was next covered with a layer of gutta-percha. The cavity was then filled with amalgam.

On January 25, 1872, this case was opened. On removing the fillings and a thin layer of decayed dentine a distinct pulp opening was found which was unmistakable. The size of it was about the twentieth of an inch and was of circular form. On the examination of this aperture a solid obstruction was found at a short distance. This was sensitive, as ordinary dentine usually is. Pressure was made on this point with considerable force upon wet cotton, which gave no pain, proving that the pulp had entirely obliterated the exposure. The sensibility of the adjacent dentine was normal.

I had previously and have since not infrequently observed the distinct step inward to the secondary dentine as was observed in this case.

To-day the same tooth presented with a pulp exposure from caries on a proximate surface at the cervix, which was capped in the usual manner I now pursue.

I bring the case before you as one evidence out of many in my experience where there has not been the least indication of pulp degeneration. It has not been uncommon for those distrusting conservative treatment of the pulp to maintain that progressive degeneracy of the pulp tissue is a necessary consequence following this mode of treatment. I may state here, it has been rare to find nodulation when subsequently the pulp has become devitalized. I am inclined to take this occasion to reiterate that, with correct precautions, pulp exposures previous to the occurrence of objective indications of pulp irritation is an entirely rational and generally successful method of treatment.

*Question.*—Will Dr. Jack give his experience relative to electric osmosis and what that experience continues to be?

*Dr. Jack.*—I have the satisfaction to state that the results continue favorable.

There is one important feature of the application of this means of giving relief from pain. This is in reference to the statement that the sensation produced by the current when the pain limit is reached is due to the evolution of heat in the tissue caused by the resistance of the dentine to the current.

In a number of instances I have made the test of applying five, ten, and fifteen chloride of silver dry cells. These are presumably of one volt each. In each case the pain limit is reached at the same degree of ampèrage. As current strength is the source of electrical energy this result is in agreement with electrical laws.

The pain limit appears with different persons at various degrees of ampèrage, according as the required resistance is varied; but more particularly for the reason that thermal irritation of dentine is a very variable condition. In other words, the temperature sense of the dentine differs in individuals.

The foregoing statements indicate that high voltage is not required, as only a certain degree of strength of current in milliampères will be tolerated. This can generally be forced into the tissue by a very small voltage rate. If the initial voltage be comparatively high, the resistance must be so great as to reduce the actual voltage to a tolerable rate.

There is one other important consideration connected with the application of electricity in cataphoresis, which is that the resistance of the controller at the highest part should be so extreme that no impulse is given at the commencement of the administration. At present I have in use a Willms controller, having a resistance at first of over three hundred thousand ohms. With this there is no initial impulse when the circuit is closed. With the former controller, having a resistance stated to be of ninety thousand ohms, some persons would feel the first contact with five volts of current. This question of the impulse of current is well elucidated by Dr. Price in an article to appear in the February number of the *Dental Cosmos.* There is nothing more clear from my experience than the necessity for minimizing the electro-motive force at the commencement of the administration. The great value of this means of treatment is in some danger of being prejudiced by using high initial voltage with insufficient resistance. The apparent requirements are low initial voltage, probably ranging between five and twenty, with resistance commencing at two hundred thousand ohms or over. The purpose of this very high resistance being, as previously indicated, to avoid the initial impulse.

*Dr. Roberts.*—Dr. Jack's illustration simply proves that the voltage only is necessary to overcome the resistance of circuit; that the number of volts that we use does not make pain or lessen the pain. It is the amount of current or the ampèrage which passes through which does the work, and the voltage is only necessary to force the current through the resistance. If the five volts will produce the result, that is all that is necessary, or fifteen volts, if you have to use that ampèrage, there is no more pain when the same amount of current is going through it. It is not the voltage which gives pain, it is the ampèrage, in connection with the controller. I believe that a controller based upon the resistance and taken out is based upon the wrong principle. The voltage should be increased or decreased correctly and not the resistance, and that can be done.

*Dr. Jack.*—There is another point of importance not directly stated in this discussion,—that is, while some persons will indicate the pain limit being reached at a given point of the controller with an initial voltage of five cells, another person may not indicate the pain limit at the same point of the controller with an initial voltage of fifteen cells. In the one case the ampèrage will be less usually than three-twentieths milli, in the other three- or four-tenths milli.

Adjourned.

H. H. Burchard, M.D., D.D.S.,
*Editor of the Academy of Stomatology.*

